# 1-(3-Phenyl­prop-2-yn­yl)pyrrolidinium chloride

**DOI:** 10.1107/S1600536809042329

**Published:** 2009-10-23

**Authors:** Tao Pang, Hui Lu, Tao Pang

**Affiliations:** a Key Laboratory of Pesticides and Chemical Biology of the Ministry of Education, College of Chemistry, Central China Normal University, Wuhan 430079, People’s Republic of China

## Abstract

The title compound C_13_H_16_N^+^·Cl^−^, an achiral salt, was synthesized by a three-component coupling reaction in the presence of copper(I) iodide. The configuration of five-membered ring is close to an envelope conformation. The crystal structure is stabilized by inter­molecular C—H⋯Cl and N—H⋯Cl inter­actions.

## Related literature

For the preparation of the title compound, see: Nilsson *et al.* (1992 ▶). For background to propargylamines, see: Dyker (1999 ▶); Hattori *et al.* (1993 ▶); Konishi *et al.* (1990 ▶). 
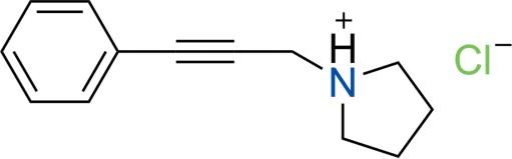


## Experimental

### 

#### Crystal data

C_13_H_16_N^+^·Cl^−^
*M* *_r_* = 221.72
Monoclinic, 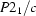

*a* = 10.9504 (2) Å
*b* = 11.3553 (3) Å
*c* = 11.1951 (2) Å
β = 117.008 (1)°
*V* = 1240.24 (4) Å^3^
*Z* = 4
Mo *K*α radiation
μ = 0.28 mm^−1^
*T* = 298 K
0.20 × 0.10 × 0.10 mm

#### Data collection

Bruker SMART CCD area-detector diffractometer
Absorption correction: none
10560 measured reflections
2432 independent reflections
2353 reflections with *I* > 2σ(*I*)
*R*_int_ = 0.054

#### Refinement

*R*[*F* ^2^ > 2σ(*F* ^2^)] = 0.067
*wR*(*F* ^2^) = 0.158
*S* = 1.27
2432 reflections
139 parameters
1 restraint
H atoms treated by a mixture of independent and constrained refinement
Δρ_max_ = 0.35 e Å^−3^
Δρ_min_ = −0.21 e Å^−3^

### 

Data collection: *SMART* (Bruker, 2001 ▶); cell refinement: *SAINT* (Bruker, 2001 ▶); data reduction: *SAINT*; program(s) used to solve structure: *SHELXS97* (Sheldrick, 2008 ▶); program(s) used to refine structure: *SHELXL97* (Sheldrick, 2008 ▶); molecular graphics: *SHELXTL* (Sheldrick, 2008 ▶); software used to prepare material for publication: *SHELXTL*.

## Supplementary Material

Crystal structure: contains datablocks I, global. DOI: 10.1107/S1600536809042329/pb2009sup1.cif

Structure factors: contains datablocks I. DOI: 10.1107/S1600536809042329/pb2009Isup2.hkl

Additional supplementary materials:  crystallographic information; 3D view; checkCIF report

## Figures and Tables

**Table 1:** Hydrogen-bond geometry (Å, °)

*D*—H⋯*A*	*D*—H	H⋯*A*	*D*⋯*A*	*D*—H⋯*A*
C6—H6⋯Cl1^i^	0.93	2.81	3.726 (3)	170
C9—H9*A*⋯Cl1^ii^	0.97	2.61	3.547 (3)	164
N1—H1⋯Cl1^iii^	0.868 (10)	2.161 (10)	3.028 (2)	178 (3)

Symmetry codes: (i) 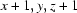; (ii) 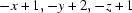; (iii) 
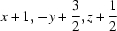
.

**Fig. 1.**
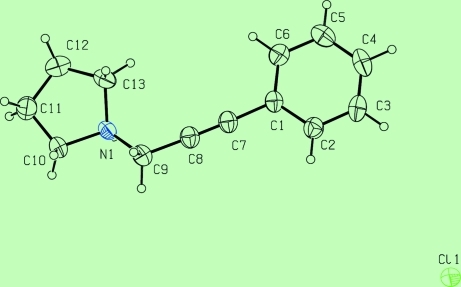
View of the molecule of (I) showing the atom-labelling scheme. Displacement ellipsoids are drawn at the 30% probability level. H atoms are represented by spheres of arbitrary radius.

## supplementary crystallographic information

### Comment

Propargylamines, which have interesting biological activities, are compounds of versatile and important synthetic intermediates. (Konishi *et al.*, 1990; Hattori *et al.*, 1993; Dyker *et al.*,1999). The reaction which a three component procedure between terminal alkynes, formaldehyde and secondary amines and give rise to the propargylamines with rapid reaction rates by the introduction of copper (I) catalysts.

Here we report the crystal structure of the title compound (Fig. 1). The length of the N1—C9 bond in this compound was found to be 1.479 Å, which is approximate with the length of ordinary N—C single bond (1.47 Å).The four carbon atoms of the five–member ring are not in the same plane, the torsion angle is 14.92 °. It was close to the envelope conformation.

X-ray analysis reveals that the crystal structure is stabilized by C—H···Cl interaction and N—H···Cl interaction.

### Experimental

The title compound was synthesized according to the literature procedure of Nilsson *et al.* (1992).

Single crystals suitable for X–ray diffraction were prepared by slow evaporation of a solution of the title compound in chloroform : methanol (20 : 1)  and adding 1d HCl at room temperature.

### Refinement

All H atoms were initially located in a difference map, but were constrained to an idealized geometry. Constrained bond lengths and isotropic displacement parameters: (C—H = 0.97 Å) and *U*_iso_(H) =1.2 *U*_eq_(C) for methylene, and (C—H = 0.93 Å) and *U*_iso_(H) =1.2*U*_eq_(C) for aromatic H atoms.

### Crystal data

**Table:**

C_13_H_16_N^+^·Cl^−^	*F*(000) = 472
*M**_r_* = 221.72	*D*_x_ = 1.187 Mg m^−^^3^
Monoclinic, *P*2_1_/*c*	Mo *K*α radiation, λ = 0.71073 Å
Hall symbol: -P 2ybc	Cell parameters from 5548 reflections
*a* = 10.9504 (2) Å	θ = 2.7–28.1°
*b* = 11.3553 (3) Å	µ = 0.28 mm^−^^1^
*c* = 11.1951 (2) Å	*T* = 298 K
β = 117.008 (1)°	Block, colorless
*V* = 1240.24 (4) Å^3^	0.20 × 0.10 × 0.10 mm
*Z* = 4	

### Data collection

**Table:**

Bruker SMART CCD area-detector diffractometer	2353 reflections with *I* > 2σ(*I*)
Radiation source: fine-focus sealed tube	*R*_int_ = 0.054
graphite	θ_max_ = 26.0°, θ_min_ = 2.1°
φ and ω scans	*h* = −13→13
10560 measured reflections	*k* = −14→14
2432 independent reflections	*l* = −13→13

### Refinement

**Table:**

Refinement on *F*^2^	Primary atom site location: structure-invariant direct methods
Least-squares matrix: full	Secondary atom site location: difference Fourier map
*R*[*F*^2^ > 2σ(*F*^2^)] = 0.067	Hydrogen site location: inferred from neighbouring sites
*wR*(*F*^2^) = 0.158	H atoms treated by a mixture of independent and constrained refinement
*S* = 1.27	*w* = 1/[σ^2^(*F*_o_^2^) + (0.053*P*)^2^ + 0.6238*P*] where *P* = (*F*_o_^2^ + 2*F*_c_^2^)/3
2432 reflections	(Δ/σ)_max_ < 0.001
139 parameters	Δρ_max_ = 0.35 e Å^−^^3^
1 restraint	Δρ_min_ = −0.21 e Å^−^^3^

### Special details

**Table:**

Geometry. All esds (except the esd in the dihedral angle between two l.s. planes) are estimated using the full covariance matrix. The cell esds are taken into account individually in the estimation of esds in distances, angles and torsion angles; correlations between esds in cell parameters are only used when they are defined by crystal symmetry. An approximate (isotropic) treatment of cell esds is used for estimating esds involving l.s. planes.
Refinement. Refinement of F^2^ against ALL reflections. The weighted R-factor wR and goodness of fit S are based on F^2^, conventional R-factors R are based on F, with F set to zero for negative F^2^. The threshold expression of F^2^ > 2sigma(F^2^) is used only for calculating R-factors(gt) etc. and is not relevant to the choice of reflections for refinement. R-factors based on F^2^ are statistically about twice as large as those based on F, and R- factors based on ALL data will be even larger.

### Fractional atomic coordinates and isotropic or equivalent isotropic displacement parameters (Å^2^)

**Table:**

	*x*	*y*	*z*	*U*_iso_*/*U*_eq_	
C1	0.6296 (3)	0.8606 (2)	0.7343 (3)	0.0493 (6)	
C2	0.4916 (3)	0.8723 (3)	0.6473 (3)	0.0620 (7)	
H2	0.4636	0.9243	0.5755	0.074*	
C3	0.3957 (3)	0.8078 (3)	0.6660 (4)	0.0735 (9)	
H3	0.3032	0.8162	0.6069	0.088*	
C4	0.4359 (4)	0.7315 (3)	0.7707 (4)	0.0774 (10)	
H4	0.3708	0.6878	0.7831	0.093*	
C5	0.5724 (4)	0.7188 (3)	0.8581 (4)	0.0734 (9)	
H5	0.5991	0.6663	0.9294	0.088*	
C6	0.6697 (3)	0.7830 (2)	0.8413 (3)	0.0590 (7)	
H6	0.7620	0.7746	0.9013	0.071*	
C7	0.7303 (3)	0.9288 (2)	0.7156 (3)	0.0551 (7)	
C8	0.8158 (3)	0.9840 (2)	0.7040 (3)	0.0570 (7)	
C9	0.9214 (3)	1.0575 (2)	0.6955 (3)	0.0581 (7)	
H9A	0.9276	1.1316	0.7410	0.070*	
H9B	0.8952	1.0748	0.6021	0.070*	
C10	1.1669 (3)	1.0766 (3)	0.7524 (3)	0.0666 (8)	
H10A	1.1679	1.0699	0.6665	0.080*	
H10B	1.1513	1.1584	0.7666	0.080*	
C11	1.2991 (3)	1.0342 (3)	0.8625 (3)	0.0759 (9)	
H11A	1.3503	1.0994	0.9190	0.091*	
H11B	1.3545	0.9971	0.8258	0.091*	
C12	1.2620 (4)	0.9460 (3)	0.9424 (3)	0.0784 (9)	
H12A	1.2790	0.8662	0.9227	0.094*	
H12B	1.3153	0.9601	1.0378	0.094*	
C13	1.1110 (3)	0.9647 (3)	0.8992 (3)	0.0640 (8)	
H13A	1.0679	0.8928	0.9081	0.077*	
H13B	1.0967	1.0265	0.9513	0.077*	
Cl1	0.03151 (7)	0.70879 (6)	0.07738 (7)	0.0545 (2)	
N1	1.0572 (2)	0.99964 (19)	0.7561 (2)	0.0491 (5)	
H1	1.051 (3)	0.9386 (17)	0.707 (2)	0.059*	

### Atomic displacement parameters (Å^2^)

**Table:**

	*U*^11^	*U*^22^	*U*^33^	*U*^12^	*U*^13^	*U*^23^
C1	0.0479 (14)	0.0489 (14)	0.0587 (15)	−0.0012 (11)	0.0308 (12)	−0.0117 (12)
C2	0.0534 (16)	0.0660 (17)	0.0676 (18)	0.0029 (13)	0.0284 (14)	0.0021 (14)
C3	0.0471 (16)	0.082 (2)	0.090 (2)	−0.0070 (15)	0.0306 (16)	−0.0131 (18)
C4	0.079 (2)	0.075 (2)	0.103 (3)	−0.0238 (18)	0.063 (2)	−0.019 (2)
C5	0.095 (3)	0.0597 (18)	0.078 (2)	−0.0041 (16)	0.051 (2)	0.0032 (15)
C6	0.0556 (16)	0.0596 (17)	0.0616 (17)	0.0044 (13)	0.0265 (14)	−0.0063 (13)
C7	0.0528 (15)	0.0557 (15)	0.0642 (17)	−0.0009 (12)	0.0329 (14)	−0.0069 (12)
C8	0.0559 (16)	0.0579 (16)	0.0628 (17)	−0.0023 (13)	0.0317 (14)	−0.0067 (13)
C9	0.0617 (17)	0.0524 (15)	0.0664 (18)	−0.0038 (13)	0.0345 (15)	−0.0002 (13)
C10	0.0595 (17)	0.0709 (19)	0.0699 (19)	−0.0164 (14)	0.0297 (15)	0.0096 (15)
C11	0.0622 (19)	0.095 (2)	0.070 (2)	−0.0112 (17)	0.0293 (17)	−0.0061 (18)
C12	0.080 (2)	0.085 (2)	0.0586 (18)	0.0083 (18)	0.0219 (17)	0.0059 (16)
C13	0.081 (2)	0.0623 (17)	0.0562 (17)	−0.0052 (15)	0.0383 (16)	0.0044 (13)
Cl1	0.0537 (4)	0.0555 (4)	0.0528 (4)	0.0018 (3)	0.0228 (3)	0.0045 (3)
N1	0.0566 (13)	0.0470 (12)	0.0498 (12)	−0.0099 (10)	0.0296 (11)	−0.0019 (9)

### Geometric parameters (Å, °)

**Table:**

C1—C2	1.383 (4)	C9—H9B	0.9700
C1—C6	1.388 (4)	C10—C11	1.492 (5)
C1—C7	1.439 (4)	C10—N1	1.501 (3)
C2—C3	1.372 (4)	C10—H10A	0.9700
C2—H2	0.9300	C10—H10B	0.9700
C3—C4	1.360 (5)	C11—C12	1.516 (5)
C3—H3	0.9300	C11—H11A	0.9700
C4—C5	1.372 (5)	C11—H11B	0.9700
C4—H4	0.9300	C12—C13	1.512 (5)
C5—C6	1.373 (4)	C12—H12A	0.9700
C5—H5	0.9300	C12—H12B	0.9700
C6—H6	0.9300	C13—N1	1.488 (3)
C7—C8	1.182 (4)	C13—H13A	0.9700
C8—C9	1.465 (4)	C13—H13B	0.9700
C9—N1	1.479 (4)	N1—H1	0.868 (10)
C9—H9A	0.9700		
			
C2—C1—C6	119.1 (3)	N1—C10—H10A	110.5
C2—C1—C7	120.7 (3)	C11—C10—H10B	110.5
C6—C1—C7	120.3 (3)	N1—C10—H10B	110.5
C3—C2—C1	120.6 (3)	H10A—C10—H10B	108.7
C3—C2—H2	119.7	C10—C11—C12	106.3 (3)
C1—C2—H2	119.7	C10—C11—H11A	110.5
C4—C3—C2	120.1 (3)	C12—C11—H11A	110.5
C4—C3—H3	120.0	C10—C11—H11B	110.5
C2—C3—H3	120.0	C12—C11—H11B	110.5
C3—C4—C5	120.2 (3)	H11A—C11—H11B	108.7
C3—C4—H4	119.9	C13—C12—C11	105.4 (3)
C5—C4—H4	119.9	C13—C12—H12A	110.7
C4—C5—C6	120.6 (3)	C11—C12—H12A	110.7
C4—C5—H5	119.7	C13—C12—H12B	110.7
C6—C5—H5	119.7	C11—C12—H12B	110.7
C5—C6—C1	119.6 (3)	H12A—C12—H12B	108.8
C5—C6—H6	120.2	N1—C13—C12	102.8 (2)
C1—C6—H6	120.2	N1—C13—H13A	111.2
C8—C7—C1	178.0 (3)	C12—C13—H13A	111.2
C7—C8—C9	176.5 (3)	N1—C13—H13B	111.2
C8—C9—N1	112.1 (2)	C12—C13—H13B	111.2
C8—C9—H9A	109.2	H13A—C13—H13B	109.1
N1—C9—H9A	109.2	C9—N1—C13	115.9 (2)
C8—C9—H9B	109.2	C9—N1—C10	112.3 (2)
N1—C9—H9B	109.2	C13—N1—C10	104.7 (2)
H9A—C9—H9B	107.9	C9—N1—H1	107 (2)
C11—C10—N1	106.1 (2)	C13—N1—H1	110 (2)
C11—C10—H10A	110.5	C10—N1—H1	106.3 (19)

### Hydrogen-bond geometry (Å, °)

**Table:**

*D*—H···*A*	*D*—H	H···*A*	*D*···*A*	*D*—H···*A*
C6—H6···Cl1^i^	0.93	2.81	3.726 (3)	170
C9—H9A···Cl1^ii^	0.97	2.61	3.547 (3)	164
N1—H1···Cl1^iii^	0.87 (1)	2.16 (1)	3.028 (2)	178 (3)

Symmetry codes: (i) *x*+1, *y*, *z*+1; (ii) −*x*+1, −*y*+2, −*z*+1; (iii) *x*+1, −*y*+3/2, *z*+1/2.
